# High-school students and self-injurious thoughts and behaviours: clues of emotion dysregulation

**DOI:** 10.1186/s13052-021-00958-0

**Published:** 2021-01-22

**Authors:** Caterina Zanus, Sara Battistutta, Renata Aliverti, Lorenzo Monasta, Marcella Montico, Luca Ronfani, Marco Carrozzi

**Affiliations:** 1 Institute for Maternal and Child Health - IRCCS “Burlo Garofolo”, Trieste, Italy; 2 Child and Adolescent Neuropsychiatry Service, Azienda Sanitaria Universitaria Friuli Centrale, Udine, Italy

**Keywords:** Suicide, Self-harm, Self-injurious, Emotion dysregulation, Adolescence

## Abstract

**Background:**

Suicide attempts and self-harm in adolescence are a major public health concern: they are among the main causes of disability-adjusted life-years worldwide, with severe long-term health consequences in terms of mental illness and psychiatric hospitalisation and a significantly increased risk of suicide. Several studies recently focused on the hypothesis that adolescents may be particularly vulnerable to emotional dysregulation and on the relation between problems with emotion regulation and suicidal and self-harming behaviours.

Italian epidemiological data about prevalence of these behaviours at the community level are lacking.

Our study aimed to estimate the prevalence of self-injurious thoughts and behaviours (SITBs) in a representative sample of community adolescents, and to examine the association between SITBs and the emotional and behavioural profiles.

**Methods:**

Anonymous self-report questionnaires were completed by 1507 students aged 11–18 years from 24 high schools in the North-eastern Italian region of Friuli Venezia Giulia. Information was collected on SITBs, on the socio-environmental context, and on the psychological profile (‘Achenbach’s YSR questionnaire 11–18, Multidimensional Test of Self-harm and Multi-Attitude Suicide Tendency Scale).

**Results:**

Overall, 11.1% of adolescents reported self-harming behaviours without suicide ideation or attempts, 6.4% declared having thought to suicide without acting a suicide attempt or self-harm, 1.4% declared having attempted suicide and really thought to take away their life. Access to health services following a suicide thought, a self-harming behaviour or suicide attempt was infrequent, particularly for suicide ideation. At the YSR, all the SITBs groups reported high scores in almost all scales, with the most evident differences in the self-harming groups in which adolescents reported significantly higher scores in all scales, both internalising and externalising. An emotion dysregulation profile was found in almost all the groups.

**Conclusions:**

This study provides us with an estimate of the prevalence of SITBs in the adolescent population and confirms the importance of further investigating the association between SITBs and emotion dysregulation. The naturalistic setting of community studies appears to be useful for studies in this field, and it allows to approach the onerous and often neglected issue of adolescent suicidality.

**Supplementary Information:**

The online version contains supplementary material available at 10.1186/s13052-021-00958-0.

## Background

Adolescence is a phase of the lifespan associated with changes across widespread biological and psychological domains, including physical, social, cognitive, and emotional. In essence, it encompasses the numerous developmental changes and foundational learning experiences that should characterise the transition from childhood to the attainment of adulthood [1]. Developmental changes during adolescence include structural and functional changes in the brain, particularly in neural systems involved in cognitive, emotional, social and motivation processes. Consequences are behavioural changes, such as increases in sensation-seeking, and a re-orientation of attention and motivation, towards peers, social evaluation, status and prestige, and sexual and romantic interests [1, 2].

The developmental trajectory from childhood to emerging adulthood is fraught with a multitude of risks and vulnerabilities. Typical elevations in emotional and physiological reactivity and greater emotional lability occur in a period of particular exposure to stressors, like changing social dynamics, higher levels of conflict with parents and disappointments and frustrations in achievement-related domains [3]. The confluence of increased exposure and perception of emotional antecedents and the elevated subjective and physiological responses to those antecedents may overwhelm the ability to regulate emotional responses effectively [1].

Developmental variations in the use of emotion regulation strategies may contribute to increase vulnerability to psychopathology [1]. The hypothesis that some adolescents may be particularly vulnerable to emotional dysregulation is suggested by the evidence that adolescence is associated with an increased incidence of both internalising and externalising symptoms [4]. Indeed, adolescence is characterised by a particularly high risk for the onset of many common forms of psychopathology (including major depression, eating disorders, substance use disorders, and some anxiety disorders) and the median age of onset for many mental disorders falls in the period of adolescence [1].

Several studies recently focused on the relationship between problems with emotion regulation and suicidal and self-harming behaviours. Self-harm is today considered a major public health concern [5]: it is one of the main causes of disability-adjusted life-years (DALYs) worldwide, with severe long-term health consequences in terms of mental illness and psychiatric hospitalisation and a significantly increased risk of suicide [6]. Over the past 45 years, suicide rates worldwide have increased by 60%, youths being the group at highest risk in a third of countries [7] making it the second most important cause of death during adolescence and young adulthood in 2013 [8]. Interestingly, community-based studies show how much self-harming behaviours are frequent among adolescents, and clinical experience suggests interpreting them from a dimensional perspective rather than a problem associated with a specific psychiatric diagnosis.

Although international variation exists, findings from many community-based studies show that around 10% of adolescents report having self-harmed, of whom some will declare some extent of suicidal intent underpinning their self-harm [5]. Presentation to hospital occurs in only about one in eight adolescents who self-harm in the community, this behaviour remaining largely hidden (at least from clinical services) at the community level [5].

Among adolescent psychiatric inpatients, the relation between emotion dysregulation and suicide ideation and attempts has been examined, and an association between perceived limited emotion regulation strategies and suicide ideation has been reported [9].

Given these considerations in this study we pursued two main objectives: 1) to explore the prevalence and characteristics of self-injurious thoughts and behaviours (SITBs) among adolescents of our Region (Friuli Venezia Giulia, Northeast Italy), investigating them at the community level; 2) to investigate the emotional and behavioural profile and perceived self-esteem of adolescents reporting SITBs, looking for signs of emotional dysregulation.

## Methods

### Participants

We planned to enrol 1500 adolescents based on the following calculations. We adopted a stratified sampling design, enrolling adolescents from three different types of high schools: 1. Licei and Teacher Training Schools (*Licei* and *Istituti Magistrali*), 2. Technical and Art Schools (*Istituti Tecnici* and *Istituti d’Arte*), 3. Training Colleges (*Istituti Professionali*). High schools were selected randomly from three comprehensive lists. Classes were also selected randomly within each school. In each school, we extracted one class per grade. Italian high schools have five grades. All pupils from selected classes were enrolled. To guarantee representativeness in each of the three strata, we calculated a sample size of 245 pupils per each type of institute, based on a preliminary study which found a prevalence of self-harm of 20% [10], and considering a confidence level of 95% (α = 0.05) and a 5% margin of error. The overall sample size would then be 735. However, considering the design effect, we decided to enrol a minimum of 1470 adolescents. With an average number of 20 pupils per class, we randomly extracted 15 schools (five per type), and five classes in each school.

### Materials

The tools used include two ad hoc questionnaires developed for this study (“Self-harming” and “Context”), and three standardised questionnaires (Achenbach’s YSR Questionnaire 11–18, Multidimensional Test of Self-Esteem, Multi-Attitude Suicide Tendency Scale). These instruments are described in detail below.

#### Self-harming questionnaire

The self-harming questionnaire was developed ad hoc. The questionnaire included three main questions: I) “*Have you ever attempted to take away your life*?”, II) “*Have you ever seriously thought about taking away your life*?”, III) “*Have you ever deliberately hurt yourself*?”

For each of these questions, the subject could answer “Yes” or “No”, and in case of an affirmative answer other questions followed to gather information on the details of the event: when it occurred, the reason for that behaviour or thought, and its consequences (if there had been any contact with a healthcare facility, which one and with what consequences).

We developed the questionnaire taking into account the suggestions emerging from the literature on community-based studies on SITBs. In this field of research, many tools exist for investigating such events. The choice of the method (validated scale, adapted scale, ad hoc checklist/questionnaire) may be influenced by many factors, sometimes language and cultural issues are involved as well. The Italian language is particularly challenging in this field as many phrases may be used to talk about suicide, each one implying or reminding quite different meanings, even more in an age-specific manner; for an adolescent, apparently similar terms like “to kill yourself”, “to take away your own life”, “to suicide” may take on clearly different meanings.

In a systematic review of current empirical studies reporting on the prevalence of non-suicidal self-harm and deliberate self-harm in adolescents [11], highlighted some methodological issues such as the lower reliability of single-item assessment vs. checklist/questionnaire-based investigation and the need to specify the period to which the behaviour refers. The authors recommend a gold-standard assessment process that would include a single item assessment that, if endorsed positively, would be followed up by an interview process to ensure the participant understands the behaviour in the same way the researcher is defining the self-injurious nature of the behaviour. In our study, to encourage the participation of the adolescents, we chose an anonymous investigation approach. We tried to make the meaning and purpose of the questions as clear as possible by preceding the administration of the questionnaires with a detailed explanation by the researcher; the presence of the researcher in the classroom assured to the adolescents the opportunity of asking for clarification.

To identify suicidal attempts we chose the expression “to take away your own life” as in the clinical practice we evaluated it was the most general and acceptable way of asking and, at the same time, it is specific enough to explore such events. We conceived the three main questions to differentiate suicidal attempts (question I), from suicidal ideation (question II) and self-harming behaviours (question III). By posing different questions and asking the subject to specify, for each case, the details about the event, we aimed to focus the attention on the event and to collect as much information as possible about it. Regarding the specific questions about the event, we chose open (free-field) answers aiming to avoid a list of possible examples that could influence the responder.

#### Context questionnaire

With the Context questionnaire, we collected socio-environmental information, such as parental formal education level and working status, size of the residence, student’s school path, and state of birth.

#### Achenbach’s YSR questionnaire 11–18 (YSR)

The YSR questionnaire is a psycho-diagnostic guidance tool frequently used both in clinical and in community-based studies.

It includes a first part about skills that the teenager feels to have, compared to his/her peers, at school, in terms of social relationships and extracurricular activities. The second part investigates the presence of emotional and behavioural problems. Answers are interpreted through the “YSR profile”, which is based on a dimensional model and structured in eight scales (Anxious/Depressed, Withdrawn/Depressed, Somatic Complaints, Social Problems, Thought Problems, Attention Problems, Rule-Breaking Behaviour, Aggressive Behaviour, Other problems), some of which are grouped in two syndromes: Internalising and Externalising.

We used the Italian translated versions of the Youth Self-Report (YSR) questionnaire for youths aged 11–18 years [12]. To investigate the presence of emotion dysregulation, we also analysed the answers by looking at the so-called “dysregulation profile” (DP). In literature, several studies have investigated this profile on the Child Behavior Checklist, Youth Self-Report, and Teacher’s Report Form (CBCL, YSR, TRF) defining it as characterised by elevated scores on the Anxious/Depressed, Attention Problems, and Aggressive Behaviour syndromes [13, 14].

Referring to some recent studies reporting on DP at YSR [15], we identified subjects with DP as those who had a T-score ≥ 67 on the anxious/depressed, attention problems, and aggressive behaviour scales of the YSR. Consistently with other studies, we considered the cut-off of 67 to include both adolescents with borderline and clinical scores.

#### Multidimensional test of self-esteem (TMA)

The questionnaire investigates the level of self-esteem of the subject [16]. Answers are grouped and coded to provide standard scores and deviations, which describe the level of self-esteem, compared to the peers’ average, in different areas. In particular, the theoretical model on which this tool is based defines six dimensions of self-esteem which identify the six scales of assessment: interpersonal relationships, environmental control competence, emotionality, school success, family life, bodily experience.

### Statistical analyses

Based on the answers to the “Self-harming questionnaire”, the prevalence of self-harming and suicidal ideations and behaviours was estimated. By analysing the three questions separately, the following groups were identified:

- *SA* (suicide attempts): subjects who reported having tried to take away their lives, but never having tried to self-harm and denying serious suicidal ideations;
- *SA + SI* (suicide attempts + ideation): subjects who reported having tried to take away their lives, thinking to suicide but never having tried to self-harm;
- *SH* (self-harming): subjects who reported having tried to self-harm but never tried neither thought to take away their lives;
- *SA + SH* (suicide attempts + self-harm): subjects who reported having hurt themselves and trying to take away their lives;
- SI + SH (suicide ideation + self-harm): subjects who reported having thought to take away their lives and having self-harmed;
- *SA + SH + SI* (suicide attempts + self-harm + suicide ideation): subjects who reported having harmed themselves and trying to take away their lives really thinking to do it;
- *SI* (suicide ideation): subjects who reported that they had seriously thought of taking their lives away but never having attempted of carrying out self-harming or suicidal acts;
- *Neg SA/SH/SI* (negative for suicide attempts/self-harm/suicide ideation): subjects who did not report any self-harming or suicidal ideations or behaviours, responding negatively to each of the three questions.

For each group, we performed a descriptive analysis of the answers given by the subjects to the other questionnaires. Socio-demographic and environmental characteristics (from the “Context” questionnaire), modes of gesture or thought, and consequences of gesture in terms of access to healthcare facilities (from the “Self-harming” questionnaire), emotional and behavioural profile (YSR questionnaire), self-esteem (TMA questionnaire).

To verify if there were significant associations between the outcomes considered and the socio-demographic and environmental variables, the emotional and behavioural profiles and self-esteem, we conducted Fisher exact 2-tailed tests and carried out a bivariate Poisson regression analysis, in which each group was compared with the Neg SA/SH/SI reference group.

A multivariate Poisson regression analysis was then performed. The analysis was performed for each group (SA + SI, SH, SA + SH, SA + SH + SI, SI) against the reference group (considering the answers to the “self-harming” questionnaire). The analysis allowed to identify variables associated with the outcome being considered. The presence of multicollinearity among independent variables was assessed with variance inflation factors (VIFs): the only variables to show some degree of collinearity were “living in a broken home” and “not living with both parents”, with VIFs ranging from 6.9 to 7.6 for both variables in the multivariate models we ran. Considering the VIFs never exceeded 10, we decided to keep both variables in the multivariate models.

Finally, we conducted a stratified Poisson regression analysis to study the heterogeneity in the association between Suicide Ideation and Attempt in adolescents who do and do not Self-Harm.

## Results

**Fig. 1:**
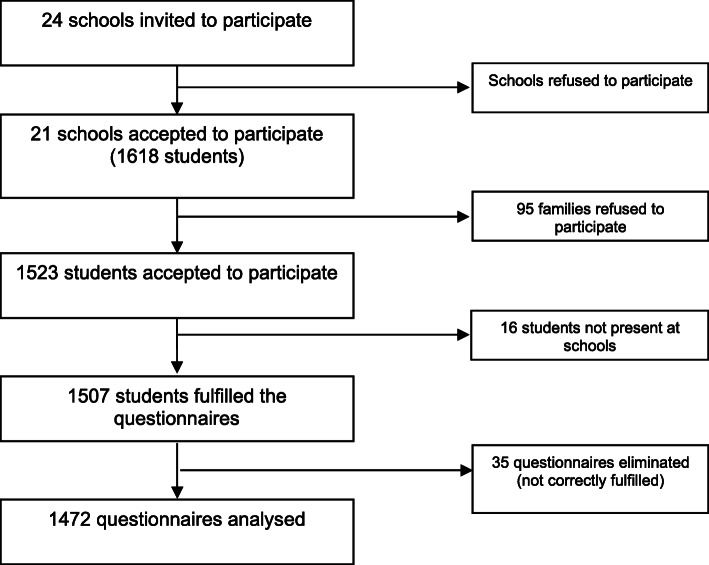
Flow chart describing how the sample of adolescents was obtained

Twenty-four high schools from Friuli Venezia Giulia Region, North-eastern Italy, were selected through a randomised system. Of these, three decided not to participate in the study. The 21 participating schools are distributed homogeneously by type of school (high schools, technical or artistic institutes and professional schools: seven schools for each type). Within each school, five classes were selected. Students who were invited to participate in the research were distributed equally from the first to fifth class, for a total of 1618 students (Fig. 1).

The teachers of the participating schools were introduced to the project through several meetings organised to formally explain the aims and methods of the research and to ask for their adhesion. After that, the adolescents answered the questionnaires anonymously, after parents’ consent, in class, in the presence of a researcher.

Of the total number of students who agreed to attend (1523), 16 were absent on the day questionnaires were distributed, so 1507 students participated in the study. Given the number of adolescents living in the Region, 75,134 for the ages of 11 to 18, of which 38,366 males and 36,798 females, the sample was considered representative of the Region (see Table 1 for the description of the general sample).

**Table 1:** Description of the general sample

**Number of subjects**	1461		
**Mean age**	16.5 years		
**Sex**	Females	815	55.8%
	Males	646	44.2%
**Type of high school**	Lyceum	586	40.1%
	Technical / Arts institute	469	32.1%
	Professional	406	27.8%
**Class repetition**	Yes	329	22.6%
	Not	1124	77.4%
	Not responding	8	
**Place of birth**	Italy	1314	90.1%
	Abroad	144	9.9%
	Not responding	3	
**Living with both parents**	Yes	1174	80.6%
	Not	283	19.4%
	Not responding	4	
**Broken home (parents separated/divorced/not living together)**	Yes	284	19.7%
	Not	1157	80.3%
	Not responding	20	
**Only child**	Yes	288	20.3%
	Not	1128	79.7%
	Not responding	45	
**Mother employed**	Yes	1051	73.0%
	Not	389	27.0%
	Not responding	21	
**Father employed**	Yes	178	12.4%
	No	1256	87.6%
	Not responding	27	

The questionnaires were delivered directly by the researchers to the students, explaining the purpose of the project and how to complete the questionnaires.

### Frequency of SITBs

**Fig. 2:**
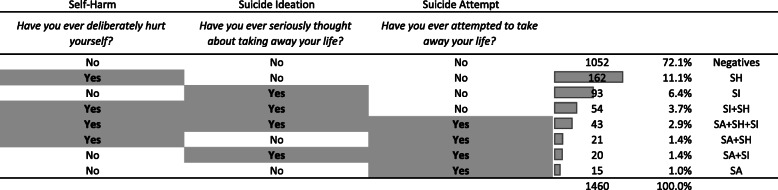
Distribution of the answers to the “Self-harming” questionnaire. Each percentage reported is on the total number of subjects

Figure 2 summarises the answers given by the adolescents to the self-harming questionnaire. Of the overall sample, some 28% answered “yes” to at least one of the three questions, while 3% answered “yes” to all of the three questions (SA + SH + SI group). Some 11.1% reported self-harming behaviors without suicide ideation or attempt (SH group). 6.4% decleared having thought to suicide without acting a suicide attempt or self-harm (SI group). 1.4% decleared having attempted suicide and really though to take away their life (SI + SA).

Suicide attempt, with or without suicide ideation, was more frequent among adolescents declaring self-harm (23% vs 3%; *p* = 0.000). However, the association between suicide ideation and suicide attempt was stronger among adolescents not declaring self-harm compared to the others (Supplemental Table 4).

### Sociodemographic data of adolescents reporting SITBs

The sociodemographic characteristics of the adolescents who declared SITBs are described in Table 2 and Supplemental Table 1. Compared to adolescents of the “Negatives” group, they showed some differences at bivariate analysis:

- students from technical and professional schools were more likely to be in the SH group if compared to students of the lyceum, but students from technical schools were less likely than those from the lyceum to be in the SI + SH gorup,
- females were proportionally more represented than males in the SI, SA + SI and SA + SI + SH groups,
- having repeated class, being born abroad and having father unemployed was significatnly more frequent in the SH group,
- being born abroad and not living with both parents was proportionally more frequent in the SI + SH group,
- not living with both parents, living in a broken home and having an unemployed father were significantly associated with being in the SA + SI + SH group.

**Table 2:** Descriptive analysis of the socio-economic characteristics of the sample

***Socio-economic variables***	Negatives	SA		SI		SH		SA + SI		SA + SH		SI + SH		SA + SI + SH	
	N (%)	N (%)	p	N (%)	p	N (%)	p	N (%)	p	N (%)	p	N (%)	p	N (%)	p
**Sex**															
Female	553 (52.6)	11 (73.3)	0.125	63 (67.7)	**0.005**	90 (55.6)	0.500	17 (85.0)	**0.005**	15 (71.4)	0.121	35 (64.8)	0.093	30 (69.8)	**0.029**
Male	499 (47.4)	4 (26.7)	“	30 (32.3)	**“**	72 (44.4)	“	3 (15.0)	**“**	6 (28.6)	“	19 (35.2)	“	13 (30.2)	**“**
**Type of High School**															
Lyceum	425 (40.4)	5 (33.3)		41 (44.1)		44 (27.2)		8 (40.0)		7 (33.3)		33 (61.1)		22 (51.2)	
Technical / Arts institute	346 (32.9)	2 (13.3)	0.469	31 (33.3)	0.805	61 (37.7)	**0.012**	2 (10.0)	0.199	8 (38.1)	0.604	9 (16.7)	**0.004**	10 (23.3)	0.148
Professional	281 (26.7)	8 (53.3)	0.153	21 (22.6)	0.417	57 (35.2)	**0.002**	10 (50.0)	0.224	6 (28.6)	0.777	12 (22.2)	0.085	11 (25.6)	0.585
**Class repetition**															
Yes	223 (21.3)	4 (26.7)	0.539	21 (22.6)	0.792	46 (28.8)	**0.041**	4 (20)	1.000	6 (28.6)	0.422	12 (23.1)	0.731	13 (30.2)	0.185
No	825 (78.7)	11 (73.3)	“	72 (77.4)	“	114 (71.3)	**“**	16 (80)	“	15 (71.4)	“	40 (76.9)	“	30 (69.8)	“
**Place of birth**															
Italy	962 (91.6)	12 (85.7)	0.335	83 (89.3)	0.438	138 (85.2)	**0.013**	18 (90)	0.682	18 (85.7)	0.414	45 (83.3)	**0.047**	38 (88.4)	0.404
Abroad	88 (8.4)	2 (14.3)	“	10 (10.8)	“	24 (14.8)	**“**	2 (10)	“	3 (14.3)	“	9 (16.7)	**“**	5 (11.6)	“
**Living with both parents**															
Yes	869 (82.8)	12 (80.0)	0.732	71 (76.3)	0.120	128 (79.0)	0.268	12 (60.0)	**0.015**	16 (76.2)	0.388	38 (70.4)	**0.027**	27 (64.3)	**0.006**
No	180 (17.2)	3 (20.0)	“	22 (23.7)	“	34 (21.0)	“	8 (40.0)	**“**	5 (23.8)	“	16 (29.6)	**“**	15 (35.7)	**“**
**Broken home**															
Yes	180 (17.3)	4 (26.7)	0.313	23 (25.0)	0.088	35 (21.9)	0.184	7 (36.8)	0.060	6 (28.6)	0.240	15 (27.8)	0.066	14 (34.2)	**0.011**
No	858 (82.7)	11 (73.3)	“	69 (75.0)	“	125 (78.1)	“	12 (63.2)	“	15 (71.4)	“	39 (72.2)	“	27 (65.9)	**“**
**Only child**															
Yes	216 (21.2)	0 (0)	0.051	13 (14.9)	0.214	29 (18.0)	0.403	1 (5.0)	0.095	7 (35.0)	0.165	13 (24.5)	0.606	9 (22.0)	0.848
No	803 (78.8)	14 (100)	“	74 (85.1)	“	132 (82.0)	“	19 (95.0)	“	13 (65.0)	“	40 (75.5)	“	32 (78.0)	“
**Mother employed**															
Yes	760 (73.4)	10 (71.4)	1.000	64 (69.6)	0.462	113 (70.6)	0.503	14 (70.0)	0.799	15 (71.4)	0.806	41 (77.4)	0.632	33 (76.7)	0.726
No	276 (26.6)	4 (28.6)	“	28 (30.4)	“	47 (29.4)	“	6 (30.0)	“	6 (28.6)	“	12 (22.6)	“	10 (23.3)	“
**Father employed**															
Yes	925 (89.0)	12 (85.7)	0.661	74 (83.2)	0.116	131 (83.4)	**0.046**	15 (75.0)	0.064	19 (100)	0.252	46 (86.8)	0.652	33 (78.6)	**0.046**
No	114 (11.0)	2 (14.3)	“	15 (16.9)	“	26 (16.6)	**“**	5 (25.0)	“	0 (0)	“	7 (13.2)	“	9 (21.4)	**“**

*p*-values of differences between each group and the reference group (“Negatives”) (two-tailed Fisher exact test)

### Frequency of access to health services by adolescents reporting SITBs

Access to health services following a suicide thought, a self-harming behaviour or suicide attempt was infrequent, particularly for suicide ideation (Table 3).

**Table 3:** Access to health services following a suicide intent, a suicide thought or a self-harming act

	SA (15)	SI (93)	SH (162)	SA + SI (20)	SA + SH (21)	SI + SH (54)	SA + SI + SH (43)	Total (408)
**Access to health service**	2/13 (15%)	3/91 (3%)	14/161 (9%)	2/20 (10%)	2/21 (10%)	5/54 (9%)	11/40 (27%)	39/400 (10%)
Did not reply to the question	2	2	1	0	0	0	3	8

### Emotional and behavioural profile of adolescents reporting SITBs

**Fig. 3:**
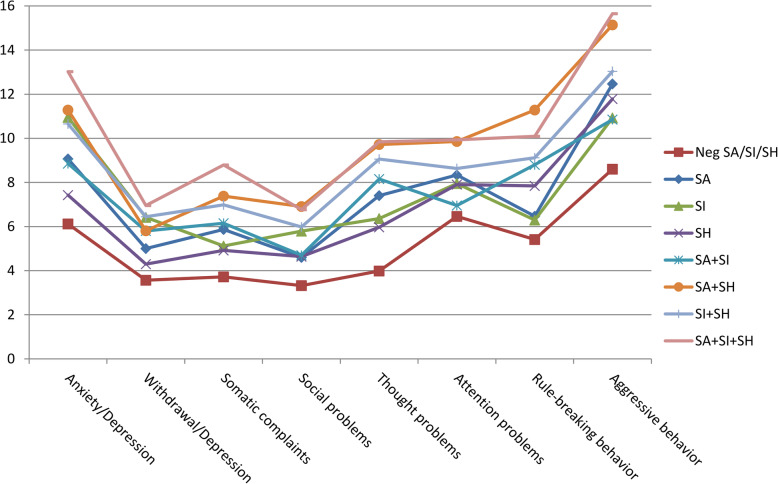
Emotional and behavioural profile, scale-specific results. The emotional and behavioural profiles of each group, based on answers to the YSR questionnaire, are represented; the mean scores for each scale of the profile are reported

**Fig. 4:**
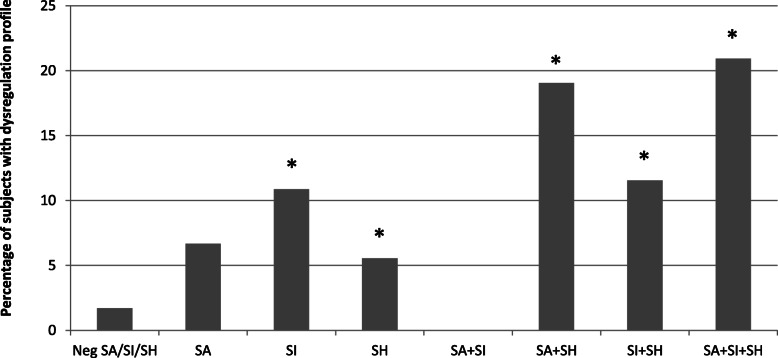
Dysregulation profile (YSR-DP). The percentage of subjects with DP is reported for each group. The group for which this percentage is significantly different compared to the “Negatives” group (NegSA/SI/SH) is identified with the asterisk sign (*)

At the analysis of the YSR profile, we found higher scores in many scales in all the SITBs groups, with the most evident differences in the SA + SH, SI + SH and SA + SI + SH groups, in which adolescents reported higher scores in all the scales, both internalising and externalising (Figs. 3 and 4). Other significant differences are detailed in Table 4 and Supplemental Table 2.

**Table 4:** Adolescents’ behavioural and emotional profiles (YSR): percentage of subjects with score in the clinical range for each of the scales of the questionnaire

YSR scales (%in clinical range)	Negatives (1050)	SA (15)	SI (92)	SH (162)	SA + SI (20)	SA + SH (21)	SI + SH (52)	SA + SI + SH (43)
Anxiety/Depression	63/1049 (6.0%)	**3 (20%; 0.061)**	**15 (16%; 0.001)**	**19 (12%; 0.011)**	2 (10%; 0.346)	**5 (24%; 0.008)**	5 (10%; 0.246)	**13 (30%; 0.000)**
Withdrawal/Depression	48/1048 (4.6%)	2 (13%; 0.154)	**23 (25%; 0.000)**	12 (7%; 0.122)	3 (15%%; 0.066)	**4 (19%; 0.016)**	**14 (27%; 0.000)**	**12 (28%; 0.000)**
Somatic complaints	44 (4.2%)	1 (7%; 0.479)	3 (3%; 1.000)	**13 (8%; 0.044)**	3 (15%; 0.054)	**4 (19%; 0.012)**	**14 (27%; 0.000)**	**17 (40% 0.000)**
Social problems	31/1048 (3.0%)	1 (7%;.370)	**16 (17%; 0.000)**	10 (6%; 0.057)	0 (0%; 1.000)	**7 (33%; 0.000)**	**8 (15%; 0.000)**	**11 (26%; 0.000)**
Thought problems	29/1047 (2.8%)	1 (7%; 0.351)	**9/91 (10%; 0.002)**	**12 (7%; 0.008)**	**4 (20%; 0.003)**	**6 (29%; 0.000)**	**15 (29%; 0.000)**	**17 (40%; 0.000)**
Attention problems	78 (7.4%)	3 (20%; 0.099)	10 (11%; 0.223)	**26 (16%; 0.001)**	1 (5%; 1.000)	**7 (33%; 0.001)**	**10 (19%; 0.006)**	**14 (33%; 0.000)**
Rule-breaking behaviour	60/1048 (5.7%)	1 (7%; 0.590)	6 (7%; 0.647)	**26 (16%; 0.000)**	**5 (25%; 0.005)**	**6 (29%; 0.001)**	**10 (19%; 0.001)**	**9 (21%; 0.001)**
Aggressive behaviour	63/1048 (6.0%)	2 (13%; 0.232)	**12 (13%; 0.015)**	**32 (20%; 0.000)**	2 (10%; 0.347)	**8 (38%; 0.000)**	**13 (25%; 0.000)**	**14 (33%; 0.000)**
Internalizing syndrome	164/1048 (15.7%)	5 (33%; 0.075)	**45 (49%; 0.000)**	**40 (25%; 0.007)**	**8 (40%; (0.009)**	**13 (62%; 0.000)**	**29 (56%; 0.000)**	**33 (77%; 0.000)**
Externalizing syndrome	206/1048 (19.7%)	6 (40%; 0.094)	**32 (35%; 0.001)**	**72 (44%; 0.000)**	**8 (40%; 0.042)**	**13 (62%; 0.000)**	**32 (66%; 0.000)**	**33 (77%; 0.000)**
Total problems	158/1048 (15.1%)	**6 (40%; 0.018)**	**40 (43%; 0.000)**	**62 (38%; 0.000)**	**9 (45%; 0.002)**	**17 (81%; 0.000)**	**35 (67%; 0.000)**	**39 (91%; 0.000)**

In parenthesis, *p*-values of differences between each group and the reference group (“Negatives”) (two-tailed Fisher exact test). Total frequencies are reported in first row, unless specified

Compared to the “Negatives” group, the percentage of adolescents with a dysregulation profile was higher in all the groups, except for the SA and SA + SI groups (Fig. 4).

### Self-esteem

**Fig. 5:**
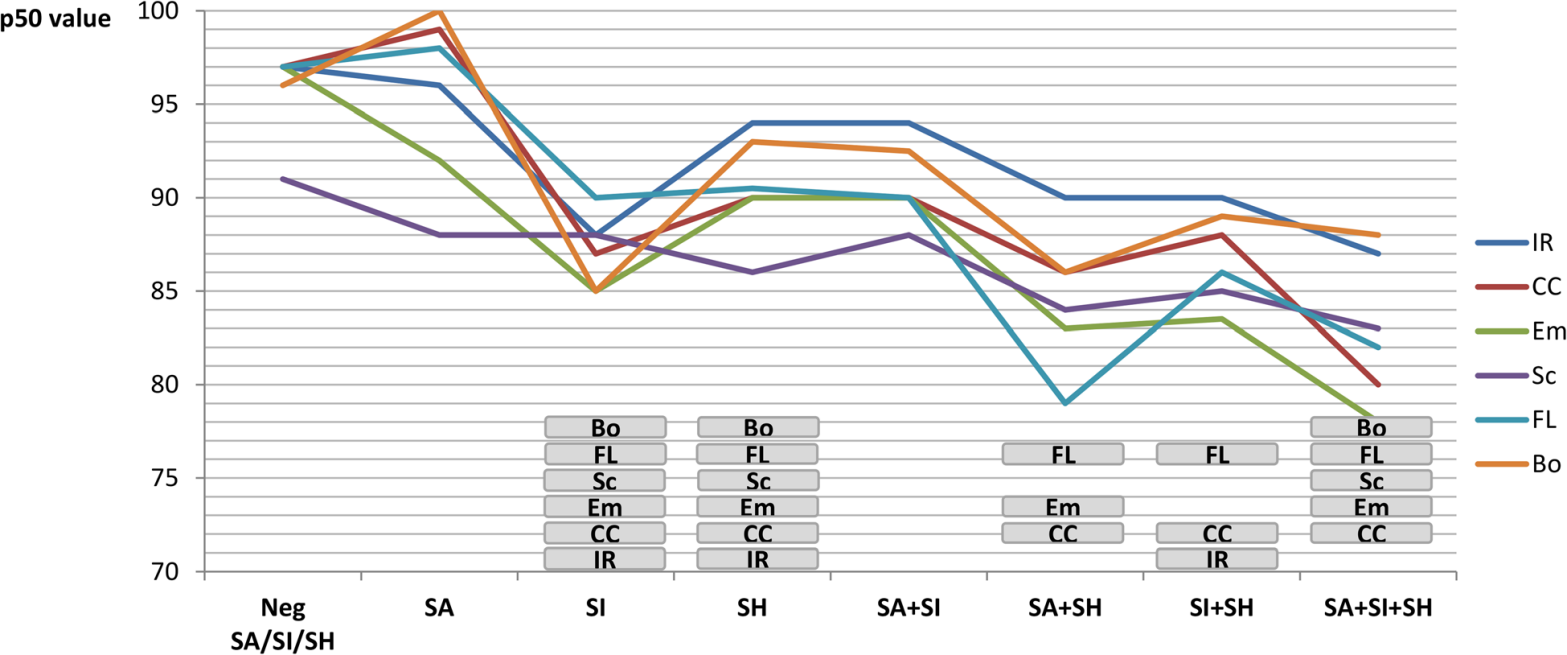
Multidimensional Test of Self-Esteem (TMA). For each group, results about different dimensions of self-esteem (IR = interpersonal relationship; CC = capacity of context control; Em = emotivity; Sc = school success; FL = family life; Bo = Body perception) are reported. Below the profiles, the dimensions with significantly lower scores, compared to the group Neg SA/SI/SH, are highlighted in grey squares

The results of the TMA questionnaire are shown in Fig. 5. Adolescents of the SI, SH and SA + SI + SH groups reported low self-esteem, compared to the “Negative” group, in all the domains. In the SA + SI + SH group, self-esteem was particularly low in the domain of emotivity.

### Results from the multivariate Poisson regression analysis

Results of the multivariate Poisson regression analyses are reported in Supplemental Tables 3a to g. Withdrawal/depression, internalizing and externalizing syndromes and female sex were significantly associated with SI, while somatic complains was inversely associated. The externalizing syndrome, being born abroad, and being a student of a technical school (vs. lyceum) was associated with self-harming behaviours. Female sex, and thought problems were significantly associated with the SA + SI group, while attention problems resulted being protective. Social problems and the internalizing syndrome were associated with the SA + SH group. Withdrawal/depression, thought problems, both internalizing and externalizing syndromes, female sex, and being a student of a lyceum were associated with the SI + SH group. Finally, both syndromes, thought problems, somatic complains, and attend the lyceum vs. a professional school were significantly associated with the SA + SI + SH group.

## Discussion

In our study, we found that, at community level, 28% of adolescents report a self-injurious thought or behavior. In particular, the 11.1% report self-harming behaviors without suicide ideation or attempt, the 6.4% decleared having thought to suicide without acting a suicide attempt or self-harm (SI group); the 1.4% decleare having attempted suicide and really though to take away their life (SI + SA). These rates are in line with those reported in the literature [5, 11] and confirm the high diffusion of SITBs among adolescents.

The overlap between self-harming and suicidal behaviours and thoughts, also confirmed by the high prevalence of suicide attempts among adolescents reporting self-harm, is consistent with the well-known co-occurrence of SITBs grounding on a complex association between non suicidal and suicidal behaviours. We found a stronger association between suicide ideation and suicide attempt among not self-harming adolescents compared to self-harming adolescents; this could be explained by the hypothesis of self-harm as a particular strategy of some adolescents to cope with their emotional difficulties while suicide attempt represents a help-seeking behaviour of others. In fact, the mechanisms of transition from suicidal thought to suicidal action are a current matter of research and the need of further longitudinal studies is clearly reported in literature [17].

The prevalence of females in SI and SA groups is consistent with literature evidences about gender differences in suicidal behaviours among adolescents [18], reporting higher risk of suicide attempt in females then in males. Nonsuicidal self-injury (NSSI) is common in both men and women. Results from clinical study report lower levels for NSSI correlates (psychopathology, suicidality) in males, suggesting the need to screen males for NSSI even when reporting comparatively less impairment [19].

Concerning the psychological profile of adolescent reporting SITBs, as our study was based on anonymous questionnaires, not allowing a clinical follow-up, we are cautious in drawing conclusions about diffusion and type of psychopathology among these adolescents. However, we consider the finding of a high prevalence of problems in emotional regulation particularly interesting.

SITBs are currently considered a major public health concern [20–23]. The association between previous SITBs and suicidal behaviour seems not to be affected by mental health or environmental aspects, suggesting that previous SITBs may be considered a risk of suicidal behaviour independently of these factors [20].

The high scores in the “total problems” YSR scale that we found in all the SITBs groups suggest a problematic emotional or behavioural profile, with a maximum intensity reached in adolescents reporting both suicide attempts and self-harm (SA + SH and SA+ SI + SH groups). The presence of both internalising and externalising problems suggests a generalised difficulty in emotions and behaviours regulation, as revealed by the high prevalence of a “dysregulation profile” in almost all the SITBs groups. Such involvement of both emotional and behavioural dimensions of psychological profile, not necessarily pathological per se but potentially resulting in dysfunctional and highly risky behaviours, seems to be more relevant than context features, when considered simultaneously in the multivariate analyses, in which sociodemographic variables seem to partly lose relevance.

The development of emotion regulation during adolescence has enjoyed a recent surge in interest. Adolescent emotional processing and regulation development seems to follow a non-linear trajectory: structural development does not always occur linearly over time nor uniformly within brain areas, meaning that different brain regions that network together to implement emotion processing and regulation develop at different rates within the same individual. This may have functional consequences, particularly for socioemotional processing and behaviour, and may at least partially explain increased emotional volatility and risk-taking at this stage in life [24, 25].

Beyond the physiological condition of emotion regulation instability in adolescence, emotion dysregulation causes the inability to manage the intensity and duration of negative emotions, to contain these emotions and to respond to interventions proposed by others for this purpose. It may reflect an early-onset limited capacity to cope with aversive affective, cognitive, and behavioural states and could represent a risk for more chronic impairment [26, 27]. In the clinical context, emotion dysregulation is considered the core deficit of Borderline Personality Disorder (BPD) [28, 29].

Emotion dysregulation is a frequently cited risk factor for both non-suicidal self-injury (NSSI) and suicidal behaviour [30, 31]. From a community study through self-report questionnaires and semi-structured interview about NSSI behaviours, Kranzler et al. found that emotion dysregulation was indirectly associated with suicide attempt frequency, via internalising symptoms and NSSI frequency, suggesting that when faced with stress or negative affect, individuals with emotion regulation deficits may be less able to tolerate these difficult emotions and more likely to internalise their distress [30].

Concerning the YSR-based “dysregulation profile” (YSR DP), in a longitudinal community sample, Deutz et al. recently demonstrated that it well describes a general vulnerability for psychopathology with emotional dysregulation at its core, intending such vulnerability not as a marker for a specific personality disorder (such as bipolar disorder), but as a potentially broad developmental precursor of personality pathology [32].

For other authors, the dysregulation profile may reflect a labile and reactive structure of psychopathology that is driven by difficulty regulating intense but transient aversive states [33].

In an Italian inpatients sample of adolescents with Non-Suicidal Self-Injury (NSSI) Ferrara et al. found a strong link between BPD diagnosis and NSSI and correlate emotionally dysregulated features of adolescents with their need to resort to NSSI for self-regulation [34].

In our study, the fact that the dysregulation profile was not significantly frequent in SA group and SA + SI group as it was in the SH groups (SH, SA + SH, SI + SH, SA + SI + SH) could be explained by the prevalence of internalising problems (polarisation of emotion profile on anxiety/depression) in adolescents reporting suicide attempts, compared to a broader difficulty in emotion and behaviour management in adolescents reporting self-harm.

The results of the SA and SA + SI groups are different for what concerns self-esteem as well: adolescents of these groups reported a quite good self-esteem at TMA, without significative differences, in any dimension of self-esteem, compared to the Negatives group. The literature generally reports low self-esteem as part of the internalising syndrome [35]. From a recent meta-analysis of longitudinal studies about the relationship between low self-esteem and suicide attempts in young people (12–26 years old), a low level of self-esteem resulted in a risk factor for suicide attempts in adolescents/young adults [36]. Interpreting our results, we have to take into account that they concern perceived self-esteem, based on an anonymous self-report questionnaire not objectivated by a structured clinical evaluation. In this sense, we read the results of self-esteem from our study as supplementary information about adolescents’ self-description instead of a pure evaluation of self-esteem. We wondered if the apparently good self-esteem of adolescents declaring suicide attempts could signify a sort of weakness in their ability to evaluate themselves and to differentiate their competences in different life domains, this being part of an internalising tendency instead of a complete internalising syndrome. In the same perspective, it is interesting that the SA + SI + SH group reported the lowest level of self-esteem in the emotivity dimension, as another possible manifestation of their inability in the emotions’ domain together with a good awareness of such inability. In fact, a frequent motivation of adolescents who self-harm is to find relief from intense emotional states, and they usually mentalise it by themselves.

Another relevant finding of our study concerns the access to health services by adolescents reporting SITBs. This result would confirm that such behaviours frequently remain hidden at the community level, seldom presenting to the hospital or coming to medical attention. We can imagine that it was an act without physical consequences needing medical consultation; this hypothesis would confirm the suggestion, from the clinical experience, that the act becomes truly visible only when it is actually harmful. Considered together, the answers “yes, I tried, or I thought about it” and “no, there wasn’t any consequence” could be translated as the experience of a failed request for help, poorly formulated and/or not sufficiently heard or adequately understood.

We are aware of some limitations of our study. Firstly, as we adopted anonymous questionnaires, we could not follow-up nor deepen the psycho-diagnostic evaluation. Secondly, we only collected data on adolescents who presented at school, potentially missing some others. We know that the categorisation in many groups may have weakened some comparisons, due to the sparsity of some groups, such as the SA group. However, we decided to maintain the subdivision, with the intention of enhancing the different meaning of the questions and answers allowed by the ad hoc created questionnaire. We think that the main strength of the study is its epidemiological relevance, as we could analyse a representative sample of the region. To date, this is the only epidemiologic study on SITBs on the Italian adolescent population. We used well-established instruments to analyse the psychological profile of adolescents, such as YSR and TMA, and, maybe favoured by anonymity, the participation was high.

In conclusion, the results of this study provided us with an estimate of the prevalence of SITBs in the adolescent population and confirmed the importance of further investigating the association between SITBs and emotion dysregulation. Interviewed at school in their daily life context, a so-called “naturalistic setting”, adolescents accept to answer on issues related to self-injurious thoughts and behaviours, declare to think or have thought to suicide or self-harm and describe their emotions and behaviours as they perceived them.

It remains questionable and hard to define to what extent the association of SITBs and emotion dysregulation could represent a transitory accentuation of a physiological phase of adolescence or disclose an actual psychopathological risk. However, the great diffusion of such problems and the well-known associated increase of suicidal risk keeps the alarm high and confirms the relevance and need to pursue and deepen studies in this field.

The naturalistic setting of community studies appears to be useful for researches in this field, allowing to approach the onerous and often neglected issue of adolescent suicidality.

## Supplementary Information

**Additional file 1.**

## Data Availability

Informed Consent did not comprise the sharing of data with different partners. However, if required, data will be shared under research protocols approved by the Institutional Review Board of our Institute.

## Abbreviations

AD: Attraction to death
AL: Attraction to life
CBCL: Child Behavior Checklist
DALYs: Disability-adjusted life-years
DP: Dysregulation profile
MAST: Multi-Attitude Suicide Tendency
Neg SA/SH/SI: Negative for suicide attempts/self-harm/suicide ideation)
RD: Repulsion by death
RL: Repulsion by life
SA: Suicide attempts
SA + I: Suicide attempts + ideation
SH: Self-harming
SI: Suicide ideation
SITBs: Self-injurious thoughts and behaviours
TRF: Teacher’s Report Form
YSR: Youth Self-Report.
