# Supplementary material for: High-school students and self-injurious thoughts and behaviours: clues of emotion dysregulation
Source: Ital J Pediatr. 2021 Jan 22;47:14. doi: 10.1186/s13052-021-00958-0 (PMC7821399; doi:10.1186/s13052-021-00958-0)
Supplement: Supplementary file 1 — Additional file 1. [file 13052_2021_958_MOESM1_ESM.docx]

**Additional File of “High-school students and self-injurious thoughts and behaviours: clues of emotion dysregulation”**

**Supplemental Table 1. Bivariate Poisson regression analysis on the association between the clinical range for each of the scales of the questionnaire vs. negatives and the socio-economic characteristics of the sample**

| ***Socio-economic variables*** | | **SA** | | **SI** | | **SH** | | **SA + SI** | | **SA + SH** | | **SI + SH** | | **SA + SI + SH** | |
| --- | --- | --- | --- | --- | --- | --- | --- | --- | --- | --- | --- | --- | --- | --- | --- |
|  |  | **IRR (95% CI)** | **p** | **IRR (95% CI)** | **p** | **IRR (95% CI)** | **p** | **IRR (95% CI)** | **p** | **IRR (95% CI)** | **p** | **IRR (95% CI)** | **p** | **IRR (95% CI)** | **p** |
| **Sex (ref. Male)** | Female | 2.5  (0.8-7.8) | 0.122 | 1.9  (1.2-3.0) | **0.006** | 1.1  (0.8-1.6) | 0.478 | 5.1  (1.5-17.6) | **0.010** | 2.3  (0.9-5.9) | 0.095 | 1.7  (0.9-2.9) | 0.081 | **2.1  (1.1-4.0)** | **0.030** |
| **Type of High School (ref. Lyceum)** | Technical / Arts institute | 0.5 (0.1-2.5) | 0.397 | 0.9 (0.6-1.5) | 0.766 | **1.7 (1.1-2.6)** | **0.011** | 0.3 (0.1-1.46) | 0.137 | 1.4  (0.5-3.9) | 0.516 | **0.3 (0.2-0.7)** | **0.004** | 0.6 (0.3-1.2) | 0.133 |
|  | Professional | 2.4  (0.8-7.5) | 0.124 | 0.8 (0.4-1.3) | 0.360 | **2.0 (1.3-3.0)** | **0.002** | 1.9 (0.7-4.8) | 0.185 | 1.3 (0.4-3.9) | 0.644 | 0.5 (0.3-1.1) | 0.084 | 0.8 (0.4-1.6) | 0.459 |
| **Class repetition (ref. No)** | Yes | 1.3 (0.4-4.2) | 0.614 | 1.1 (0.6-1.8) | 0.769 | **1.5 (1.0-2.2)** | **0.035** | 0.9 (0.3-2.8) | 0.890 | 1.5 (0.6-3.9) | 0.423 | 1.1 (0.6-2.2) | 0.758 | 1.6 (0.8-3.1) | 0.166 |
| **Place of birth (ref. Italy)** | Abroad | 1.8 (0.4-8.2) | 0.437 | 1.3 (0.7-2.6) | 0.435 | **1.9 (1.2-3.1)** | **0.009** | 1.2 (0.3-5.3) | 0.796 | 1.8 (0.5-6.3) | 0.344 | **2.2  (1.0-4.6)** | **0.040** | 1.4 (0.6-3.7) | 0.457 |
| **Living with both parents (ref. Yes)** | No | 1.2 (0.3-4.3) | 0.773 | 1.5 (0.9-2.5) | 0.118 | 1.3 (0.9-1.9) | 0.235 | 3.2 (1.3-8.0) | 0.012 | 1.5 (0.5-4.2) | 0.428 | **2.0 (1.1-3.7)** | **0.022** | **2.7 (1.4-5.1)** | **0.003** |
| **Broken home (ref. No)** | Yes | 1.7 (0.5-5.5) | 0.351 | 1.6 (0.9-2.6) | 0.069 | 1.3 (0.9-2.0) | 0.165 | **2.8 (1.1-7.2)** | **0.034** | 1.9 (0.7-5.0) | 0.188 | 1.8 (0.9-3.4) | 0.054 | **2.5 (1.3-4.8)** | **0.008** |
| **Only child (ref. No)** | Yes | - | - | 0.7 (0.4-1.2) | 0.170 | 0.8 (0.5-1.3) | 0.355 | 0.2 (0.03-1.5) | 0.113 | 2.0 (0.8-5.1) | 0.144 | 1.2 (0.6-2.3) | 0.565 | 1.0  (0.5-2.2) | 0.908 |
| **Mother employed (ref. Yes)** | No | 1.1 (0.3-3.5) | 0.871 | 1.2 (0.8-1.9) | 0.432 | 1.1 (0.8-1.7) | 0.469 | 1.2 (0.4-3.1) | 0.737 | 1.1 (0.4-2.9) | 0.843 | 0.8 (0.4-1.6) | 0.520 | 0.8 (0.4-1.7) | 0.623 |
| **Father employed (ref. Yes)** | No | 1.4 (0.3-6.1) | 0.695 | 1.6 (0.9-3.0) | 0.097 | 1.6 (1.0-2.6) | **0.044** | 2.7 (0.9-7.6) | 0.058 | - | - | 1.2 (0.5-2.8) | 0.614 | **2.2 (1.0-4.7)** | **0.041** |

*Missing values in the table are because of a zero frequency in a cell of the contingency table.*

**Supplemental Table 2. Bivariate Poisson regression analysis on the association between positivity to different SITBs vs. negatives and the positives to the YRS scales considered.**

| YSR scales in clinical range | SA | SI | SH | SA + SI | SA + SH | SI + SH | SA+SI+SH |
| --- | --- | --- | --- | --- | --- | --- | --- |
| Anxiety/Depression | **3.912 (1.076-14.221) p=0.038** | **3.049 (1.658-5.605) p=0.000** | **2.079 (1.209-3.576) p=0.008** | 1.739 (0.395-7.661) p=0.465 | **4.891 (1.736-13.782) p=0.003** | 1.665 (0.640-4.333) p=0.296 | **6.782 (3.372-13.642) p=0.000** |
| Withdrawal/Depression | 2 (13%; 0.154) | **23 (25%; 0.000)** | 12 (7%; 0.122) | 3 (15%%; 0.066) | **4 (19%; 0.016)** | **14 (27%; 0.000)** | **12 (28%; 0.000)** |
| Somatic complaints | 1 (7%; 0.479) | 3 (3%; 1.000) | **13 (8%; 0.044)** | 3 (15%; 0.054) | **4 (19%; 0.012)** | **14 (27%; 0.000)** | **17 (40% 0.000)** |
| Social problems | 1 (7%;.370) | **16 (17%; 0.000)** | 10 (6%; 0.057) | 0 (0%; 1.000) | **7 (33%; 0.000)** | **8 (15%; 0.000)** | **11 (26%; 0.000)** |
| Thought problems | 1 (7%; 0.351) | **9/91  (10%; 0.002)** | **12 (7%; 0.008)** | **4 (20%; 0.003)** | **6 (29%; 0.000)** | **15 (29%; 0.000)** | **17 (40%; 0.000)** |
| Attention problems | 3 (20%; 0.099) | 10 (11%; 0.223) | **26 (16%; 0.001)** | 1 (5%; 1.000) | **7 (33%; 0.001)** | **10 (19%; 0.006)** | **14 (33%; 0.000)** |
| Rule-breaking behaviour | 1 (7%; 0.590) | 6 (7%; 0.647) | **26 (16%; 0.000)** | **5 (25%; 0.005)** | **6 (29%; 0.001)** | **10 (19%; 0.001)** | **9 (21%; 0.001)** |
| Aggressive behaviour | 2 (13%; 0.232) | **12 (13%; 0.015)** | **32 (20%; 0.000)** | 2 (10%; 0.347) | **8 (38%; 0.000)** | **13 (25%; 0.000)** | **14 (33%; 0.000)** |
| Internalizing syndrome | 5 (33%; 0.075) | **45 (49%; 0.000)** | **40 (25%; 0.007)** | **8 (40%; (0.009)** | **13 (62%; 0.000)** | **29 (56%; 0.000)** | **33 (77%; 0.000)** |
| Externalizing syndrome | 6 (40%; 0.094) | **32 (35%; 0.001)** | **72 (44%; 0.000)** | **8 (40%; 0.042)** | **13 (62%; 0.000)** | **32 (66%; 0.000)** | **33 (77%; 0.000)** |
| Total problems | **6 (40%; 0.018)** | **40 (43%; 0.000)** | **62 (38%; 0.000)** | **9 (45%; 0.002)** | **17 (81%; 0.000)** | **35 (67%; 0.000)** | **39 (91%; 0.000)** |

*Figures reported are Rate Ratios with 95% confidence intervals in parenthesis, and p-values.*

**Supplemental Table 3a. Multivariate Poisson regression analysis on the association between suicide attempts (SA) vs. negatives and YRS scales considered and socio-demographic variables.**

| Covariates | Suicide Attempt (SA) |
| --- | --- |
| YSR Anxiety/Depression | 1.597 (0.249-10.263), p=0.622 |
| YSR Withdrawal/Depression | 2.817 (0.331-23.985), p=0.343 |
| YSR Somatic complaints | 0.876 (0.072-10.625), p=0.917 |
| YSR Social problems | 0.818 (0.064-10.389), p=0.877 |
| YSR Thought problems | 1.918 (0.144-25.614), p=0.622 |
| YSR Attention problems | 1.257 (0.216-7.305), p=0.799 |
| YSR Rule-breaking behaviour | 0.643 (0.058-7.161), p=0.720 |
| YSR Aggressive behaviour | 1.101 (0.150-8.100), p=0.925 |
| YSR Internalizing syndrome | 1.057 (0.173-6.445), p=0.952 |
| YSR Externalizing syndrome | 3.149 (0.768-12.913), p=0.111 |
| Female sex | 3.786 (0.894-16.032), p=0.071 |
| Technical / Arts institute vs. Lyceum | 0.573 (0.098-3.335), p=0.535 |
| Professional vs. Lyceum | 2.384 (0.615-9.244), p=0.209 |
| Class repetition | 0.892 (0.215-3.698), p=0.875 |
| Place of birth abroad | 1.646 (0.305-8.886), p=0.562 |
| Not living with both parents | 0.112 (0.008-1.563), p=0.104 |
| Broken home | 6.821 (0.659-70.582), p=0.107 |
| Mother unemployed | 1.11 (0.314-3.928), p=0.872 |
| Father unemployed | 1.403 (0.284-6.924), p=0.677 |

*Figures reported are Rate Ratios with 95% confidence intervals in parenthesis, and p-values.*

**Supplemental Table 3b. Multivariate Poisson regression analysis on the association between suicide ideation (SI) vs. negatives and YRS scales considered and socio-demographic variables.**

| Covariates | Suicide Ideation (SI) |
| --- | --- |
| YSR Anxiety/Depression | 0.937 (0.411-2.135), p=0.877 |
| YSR Withdrawal/Depression | **3.146 (1.462-6.774), p=0.003** |
| YSR Somatic complaints | **0.230 (0.059-0.892), p=0.034** |
| YSR Social problems | 2.343 (0.975-5.629), p=0.057 |
| YSR Thought problems | 2.259 (0.769-6.634), p=0.138 |
| YSR Attention problems | 0.459 (0.181-1.162), p=0.100 |
| YSR Rule-breaking behaviour | 0.529 (0.160-1.751), p=0.297 |
| YSR Aggressive behaviour | 1.096 (0.417-2.882), p=0.853 |
| YSR Internalizing syndrome | **3.687 (1.896-7.171), p=0.000** |
| YSR Externalizing syndrome | **2.624 (1.382-4.983), p=0.003** |
| Female sex | **1.82 (1.041-3.181), p=0.036** |
| Technical / Arts institute vs. Lyceum | 0.944 (0.524-1.700), p=0.848 |
| Professional vs. Lyceum | 0.637 (0.316-1.286), p=0.208 |
| Class repetition | 1.314 (0.686-2.517), p=0.410 |
| Place of birth abroad | 0.879 (0.355-2.174), p=0.780 |
| Not living with both parents | 1.287 (0.253-6.552), p=0.761 |
| Broken home | 1.372 (0.280-6.714), p=0.696 |
| Only child | 0.815 (0.419-1.585), p=0.547 |
| Mother unemployed | 1.296 (0.748-2.246), p=0.355 |
| Father unemployed | 0.935 (0.430-2.035), p=0.866 |

*Figures reported are Rate Ratios with 95% confidence intervals in parenthesis, and p-values.*

**Supplemental Table 3c. Multivariate Poisson regression analysis on the association between self-harming (SH) vs. negatives and YRS scales considered and socio-demographic variables.**

| Covariates | Self-Harm (SH) |
| --- | --- |
| YSR Anxiety/Depression | 1.421 (0.719-2.807), p=0.312 |
| YSR Withdrawal/Depression | 1.056 (0.459-2.429), p=0.899 |
| YSR Somatic complaints | 0.891 (0.399-1.990), p=0.778 |
| YSR Social problems | 1.624 (0.670-3.935), p=0.283 |
| YSR Thought problems | 1.562 (0.689-3.541), p=0.285 |
| YSR Attention problems | 1.153 (0.630-2.110), p=0.644 |
| YSR Rule-breaking behaviour | 1.385 (0.713-2.693), p=0.336 |
| YSR Aggressive behaviour | 1.499 (0.780-2.880), p=0.224 |
| YSR Internalizing syndrome | 1.178 (0.657-2.110), p=0.583 |
| YSR Externalizing syndrome | **2.190 (1.370-3.500), p=0.001** |
| Female sex | 1.300 (0.889-1.901), p=0.176 |
| Technical / Arts institute vs. Lyceum | **1.616 (1.031-2.533), p=0.036** |
| Professional vs. Lyceum | 1.539 (0.947-2.5), p=0.082 |
| Class repetition | 1.101 (0.715-1.695), p=0.663 |
| Place of birth abroad | **2.156 (1.275-3.643), p=0.004** |
| Not living with both parents | 0.787 (0.264-2.342), p=0.667 |
| Broken home | 1.315 (0.451-3.837), p=0.616 |
| Only child | 0.864 (0.541-1.381), p=0.541 |
| Mother unemployed | 1.001 (0.668-1.501), p=0.995 |
| Father unemployed | 1.653 (0.993-2.753), p=0.053 |

*Figures reported are Rate Ratios with 95% confidence intervals in parenthesis, and p-values.*

**Supplemental Table 3d. Multivariate Poisson regression analysis on the association between suicide attempts + ideation (SA + SI) vs. negatives and YRS scales considered and socio-demographic variables.**

| Covariates | Suicide Attempt + Suicide Ideation (SA + SI) |
| --- | --- |
| YSR Anxiety/Depression | 0.807 (0.133-4.910), p=0.816 |
| YSR Withdrawal/Depression | 1.426 (0.236-8.629), p=0.699 |
| YSR Somatic complaints | 0.646 (0.073-5.685), p=0.694 |
| YSR Thought problems | **27.460 (3.034-248.526), p=0.003** |
| YSR Attention problems | **0.055 (0.004-0.835), p=0.037** |
| YSR Rule-breaking behaviour | 7.351 (0.997-54.178), p=0.050 |
| YSR Aggressive behaviour | 0.152 (0.014-1.645), p=0.121 |
| YSR Internalizing syndrome | 3.196 (0.749-13.624), p=0.116 |
| YSR Externalizing syndrome | 2.161 (0.512-9.124), p=0.294 |
| Female sex | **11.806 (2.059-67.686), p=0.006** |
| Technical / Arts institute vs. Lyceum | 0.278 (0.052-1.478), p=0.133 |
| Professional vs. Lyceum | 1.684 (0.530-5.346), p=0.377 |
| Class repetition | 0.486 (0.099-2.393), p=0.375 |
| Place of birth abroad | 1.312 (0.262-6.573), p=0.741 |
| Not living with both parents | 1.399 (0.017-113.578), p=0.881 |
| Broken home | 2.081 (0.028-154.956), p=0.739 |
| Only child | 0.230 (0.029-1.845), p=0.167 |
| Mother unemployed | 1.661 (0.523-5.270), p=0.389 |
| Father unemployed | 1.371 (0.342-5.498), p=0.656 |

*Figures reported are Rate Ratios with 95% confidence intervals in parenthesis, and p-values.*

**Supplemental Table 3e. Multivariate Poisson regression analysis on the association between suicide attempts +self-harm (SA + SH) vs. negatives and YRS scales considered and socio-demographic variables.**

| Covariates | Suicide Attempt + Self-Harm (SA + SH) |
| --- | --- |
| YSR Anxiety/Depression | 1.116 (0.299-4.170), p=0.870 |
| YSR Withdrawal/Depression | 1.107 (0.238-5.164), p=0.897 |
| YSR Somatic complaints | 1.367 (0.293-6.375), p=0.691 |
| YSR Social problems | **4.370 (1.052-18.162), p=0.042** |
| YSR Thought problems | 4.074 (0.750-22.124), p=0.104 |
| YSR Attention problems | 0.670 (0.140-3.207), p=0.616 |
| YSR Rule-breaking behaviour | 0.811 (0.151-4.358), p=0.807 |
| YSR Aggressive behaviour | 2.833 (0.610-13.165), p=0.184 |
| YSR Internalizing syndrome | **3.962 (1.080-14.536), p=0.038** |
| YSR Externalizing syndrome | 2.317 (0.605-8.875), p=0.220 |
| Female sex | 3.118 (0.936-10.382), p=0.064 |
| Technical / Arts institute vs. Lyceum | 1.501 (0.466-4.841), p=0.496 |
| Professional vs. Lyceum | 0.682 (0.170-2.738), p=0.589 |
| Class repetition | 1.545 (0.475-5.027), p=0.470 |
| Place of birth abroad | 3.314 (0.797-13.776), p=0.099 |
| Not living with both parents | 1.128 (0.032-39.365), p=0.947 |
| Broken home | 0.830 (0.024-29.097), p=0.918 |
| Only child | 2.118 (0.713-6.297), p=0.177 |
| Mother unemployed | 0.988 (0.329-2.968), p=0.983 |

*Figures reported are Rate Ratios with 95% confidence intervals in parenthesis, and p-values.*

**Supplemental Table 3f. Multivariate Poisson regression analysis on the association between suicide ideation + self-harm (SI + SH) vs. negatives and YRS scales considered and socio-demographic variables.**

| Covariates | Suicide Ideation + Self-Harm (SI + SH) |
| --- | --- |
| YSR Anxiety/Depression | 0.349 (0.098-1.239), p=0.103 |
| YSR Withdrawal/Depression | **4.256 (1.427-12.693), p=0.009** |
| YSR Somatic complaints | 1.914 (0.637-5.749), p=0.247 |
| YSR Social problems | 1.686 (0.484-5.865), p=0.412 |
| YSR Thought problems | **6.773 (2.112-21.725), p=0.001** |
| YSR Attention problems | 0.217 (0.061-0.765), p=0.017 |
| YSR Rule-breaking behaviour | 1.578 (0.487-5.108), p=0.447 |
| YSR Aggressive behaviour | 1.226 (0.404-3.718), p=0.719 |
| YSR Internalizing syndrome | **3.106 (1.234-7.821), p=0.016** |
| YSR Externalizing syndrome | **3.923 (1.658-9.28), p=0.002** |
| Female sex | **3.381 (1.496-7.642), p=0.003** |
| Technical / Arts institute vs. Lyceum | **0.241 (0.089-0.652), p=0.005** |
| Professional vs. Lyceum | **0.393 (0.159-0.970), p=0.043** |
| Class repetition | 0.710 (0.259-1.947), p=0.506 |
| Place of birth abroad | 2.268 (0.760-6.771), p=0.142 |
| Not living with both parents | 1.690 (0.107-26.627), p=0.709 |
| Broken home | 1.024 (0.067-15.56), p=0.986 |
| Only child | 1.173 (0.524-2.626), p=0.699 |
| Mother unemployed | 0.856 (0.377-1.941), p=0.710 |
| Father unemployed | 0.752 (0.240-2.360), p=0.625 |

*Figures reported are Rate Ratios with 95% confidence intervals in parenthesis, and p-values.*

**Supplemental Table 3g. Multivariate Poisson regression analysis on the association between suicide attempts + suicide ideation + self-harm (SA + SI + SH) vs. negatives and YRS scales considered and socio-demographic variables.**

| Covariates | Suicide Attempt + Suicide Ideation + Self-Harm (SA + SI + SH) |
| --- | --- |
| YSR Anxiety/Depression | 1.487 (0.505-4.378), p=0.471 |
| YSR Withdrawal/Depression | 1.595 (0.443-5.747), p=0.475 |
| YSR Somatic complaints | **3.235 (1.075-9.733), p=0.037** |
| YSR Social problems | 2.681 (0.780-9.211), p=0.117 |
| YSR Thought problems | **5.328 (1.659-17.116), p=0.005** |
| YSR Attention problems | 0.340 (0.108-1.073), p=0.066 |
| YSR Rule-breaking behaviour | 1.516 (0.506-4.538), p=0.457 |
| YSR Aggressive behaviour | 1.243 (0.441-3.501), p=0.681 |
| YSR Internalizing syndrome | **5.071 (1.683-15.285), p=0.004** |
| YSR Externalizing syndrome | **7.873 (2.782-22.282), p=0.000** |
| Female sex | 2.063 (0.819-5.194), p=0.124 |
| Technical / Arts institute vs. Lyceum | 0.480 (0.168-1.373), p=0.171 |
| Professional vs. Lyceum | **0.242 (0.077-0.761), p=0.015** |
| Class repetition | 1.904 (0.705-5.142), p=0.204 |
| Place of birth abroad | 2.367 (0.659-8.508), p=0.187 |
| Not living with both parents | 2.022 (0.132-30.857), p=0.613 |
| Broken home | 1.008 (0.069-14.735), p=0.996 |
| Only child | 0.948 (0.352-2.553), p=0.916 |
| Mother unemployed | 0.534 (0.189-1.506), p=0.236 |
| Father unemployed | 1.634 (0.487-5.488), p=0.427 |

*Figures reported are Rate Ratios with 95% confidence intervals in parenthesis, and p-values.*

**Supplemental Table 4. Association between Suicide Intents (SI) and Suicide Attempts (SA) in adolescents who declared not to Self-Harm and to Self-Harm (SH), and analysis of the heterogeneity between these two groups.**

|  | No Self-Harm | | | | Self-Harm | | | |
| --- | --- | --- | --- | --- | --- | --- | --- | --- |
|  | No Suicide Ideation | | Suicide Ideation | | No Suicide Ideation | | Suicide Ideation | |
| No Suicide Attempt | 1052 | 98.6% | 93 | 82.3% | 162 | 88.5% | 54 | 55.7% |
| Suicide Attempt | 15 | 1.4% | 20 | 17.7% | 21 | 11.5% | 43 | 44.3% |
| Total | 1067 | 100% | 113 | 100% | 183 | 100% | 97 | 100% |

*Results of the stratified Poisson regression: in the No Self-Harm group IRR=12.6 (95% CI 6.45-24.59; p=0.000); in the Self-Harm group IRR=3.86 (95% CI 2.29-6.51; p=0.000). Test of homogeneity (M-H): chi2(1) = 8.817, Pr>chi2 = 0.0030.*
